# Exercise as Modulator of Brain-Derived Neurotrophic Factor (BDNF) in Children: A Systematic Review of Randomized Controlled Trials

**DOI:** 10.3390/life15071147

**Published:** 2025-07-21

**Authors:** Markel Rico-González, Daniel González-Devesa, Carlos D. Gómez-Carmona, Adrián Moreno-Villanueva

**Affiliations:** 1 Department of Didactics of Music, Plastic and Body Expression, University of the Basque Country, UPV-EHU, 48940 Leioa, Spain; markel.rico@ehu.eus; 2 Research Group on Physical Activity, Education and Health (GIAFES), Catholic University of Ávila, 05005 Ávila, Spain; 3 Research Group in Training, Physical Activity and Sports Performance (ENFYRED), Department of Music, Plastic and Body Expression, University of Zaragoza, 44003 Teruel, Spain; 4 Research Group in Training Optimization and Sports Performance (GOERD), University of Extremadura, 10003 Caceres, Spain; 5 BioVetMed & SportSci Research Group, University of Murcia, 30100 Murcia, Spain; adrian.moreno@ui1.es; 6 Faculty of Health Science, University Isabel I, 09003 Burgos, Spain

**Keywords:** BDNF, blood, biomarker, learning, plasticity

## Abstract

Background: Brain-derived neurotrophic factor (BDNF) plays a pivotal role in neuroplasticity and cognitive development. While exercise has been shown to modulate BDNF levels in adults, evidence in children remains limited and heterogeneous. Methods: A systematic review was conducted following PRISMA guidelines to examine randomized controlled trials investigating exercise effects on BDNF in children aged 5–12 years. The databases searched included FECYT, PubMed, SPORTDiscus, ProQuest Central, SCOPUS, and Cochrane Library through June 2025. Study quality was assessed using the PEDro scale. Results: Five randomized controlled trials (N = 385 participants) met inclusion criteria. Two studies (40%) demonstrated significant BDNF increases following exercise interventions. Successful interventions were characterized by neuromotor activities or martial arts programs, training frequencies ≥ 3 sessions/week, durations ≥ 12 weeks, and healthy participant populations. Methodological quality was mostly fair, with four studies rated as fair and one as good. Conclusions: Structured physical exercise may enhance BDNF levels in healthy children, with neuromotor activities and martial arts showing particular promise. However, children with overweight/obesity may require modified intervention approaches. The evidence supports the implementation of cognitively engaging physical activities in educational settings to optimize brain health during critical developmental periods, though larger standardized trials are needed to strengthen these preliminary findings.

## 1. Introduction

Brain-derived neurotrophic factor (BDNF) is one of the most abundant neurotrophic factors in the central nervous system and plays a pivotal role in synaptic homeostasis and neuronal plasticity [1]. Upon binding to its cognate high-affinity receptor, tropomyosin receptor kinase B (TrkB), BDNF concomitantly activates the PI3K/Akt, MAPK/ERK, and PLC-γ signaling cascades, which collectively regulate cell survival, neurogenesis, synaptogenesis, and long-term potentiation, processes that underlie memory consolidation and learning [1]; BDNF synthesis is dynamic across the lifespan and is highly sensitive to environmental stimuli, notably physical activity and stress [1,2].

Childhood (approximately 5–12 years) constitutes a critical period because circulating BDNF levels appear to be modulated by the hormonal milieu. Age and sex have specific effects on both stored and circulating blood BDNF [3]. Increasing evidence indicates that BDNF/TrkB signaling is essential for learning and memory [4]. Conversely, dysregulation of synaptic plasticity proteins, particularly BDNF, has been reported in children with neurodevelopmental disorders such as ADHD and ASD [5,6], as well as in populations with cognitive impairment and psychiatric disorders [7,8]. Lifestyle factors, including diet, sleep, and, notably, physical exercise, are associated with higher BDNF levels [9], highlighting the potential of non-pharmacological strategies to optimize this neurotrophin. Diets rich in polyphenols, phenolic acids, and related compounds appear to raise BDNF levels significantly [10]. Conversely, Giece et al. [11] suggest sleep as a key mediator at the connection between stress and BDNF.

The World Health Organization recommends that children and adolescents engage in at least 60 min of moderate to vigorous physical activity each day [12]. Additionally, it advises that vigorous-intensity aerobic activities, as well as those that strengthen muscle and bone, should be incorporated at least three days a week.

In this context, several systematic reviews have examined the effects of physical exercise interventions in adolescent and adult populations. The most recent reviews and meta-analyses consistently report that physical exercise acutely increases peripheral BDNF levels and, when performed regularly as part of a structured program, can also raise baseline concentrations. In adults aged 20–31 years, a review of 22 studies found that a single bout of high-intensity exercise (HIE) produced significantly greater BDNF increases than non-exercise or light-intensity protocols [13], consistent with findings from other systematic reviews of high-intensity interval training (HIIT) in adults [14]. Among adolescents, moderate to vigorous aerobic programs likewise appear capable of increasing BDNF [15].

Although the evidence base in this area continues to expand, current systematic reviews frequently synthesize data from both children and adolescents and do not restrict their analyses to randomized controlled trials (RCTs) [16]. To date, no systematic review has focused exclusively on RCTs that investigate the capacity of exercise to modulate BDNF levels in children. The present review therefore aims (i) to identify, critically evaluate, and synthesize evidence from RCTs assessing the effects of physical exercise interventions on BDNF concentrations in children aged 5–12 years, and (ii) to delineate the training parameters, type, intensity, duration, and frequency that are the most effective for enhancing this neurotrophin during this developmental stage. We hypothesize that structured physical exercise interventions will be associated with significant increases in circulating BDNF levels in children.

## 2. Materials and Methods

### 2.1. Experimental Approach to the Problem

The present systematic review was conducted in accordance with the Preferred Reporting Items for Systematic Reviews and Meta-Analyses (PRISMA) guidelines [17] and adhered to established guidelines for conducting systematic reviews within the domain of sport sciences [18]. The review protocol was developed with the objective of ensuring comprehensive coverage of the relevant literature while maintaining methodological rigor. This systematic review was registered in PROSPERO: CRD420251071806 (available from https://www.crd.york.ac.uk/PROSPERO/view/CRD420251071806; 1 July 2025).

### 2.2. Information Sources

The following bibliographic databases were consulted: FECYT (Web of Sciences, CCC, CIDW, KJD, MEDLINE, RSCI, and SCIELO), PubMed, SPORTDiscus, ProQuest Central, SCOPUS, and Cochrane Library. The search encompassed all published literature prior to 10 June 2025. The combination of databases was selected to ensure broad coverage of both medical and sports science literature.

### 2.3. Search Strategy

The PICO (Patient, Problem, or Population–Intervention or Exposure–Comparison, Control, or Comparator–Outcome[s]) framework was implemented in order to structure the search strategy and ensure systematic coverage of relevant literature (Table 1). In the interest of maintaining transparency, the authors were not blinded to journal names or manuscript authors. The final search string was as follows:

(preschool* OR kindergarten OR child* OR young OR childhood OR school) AND (exercise OR movement OR activity OR sport OR fitness OR aerobic OR training OR performance) AND (BDNF OR “brain-derived neurotrophic factor”) AND (“randomized controlled trial”)

### 2.4. Eligibility Criteria

The authors initiated the search string on databases and downloaded the title, authors’ names, journal, and date of all the articles that appeared in the search. Following the organization of the Excel spreadsheet, the process of removing all the duplicates was initiated. Two authors (D.G-D and C.D.G.-C.) then independently screened the titles and abstracts of the retrieved studies to assess their eligibility. Full-text versions of all potentially relevant studies were subsequently obtained. In cases where eligibility remained unclear, a third author (M.R.-G.) was consulted to resolve disagreements and make a final decision.

### 2.5. Data Extraction

A standardized data extraction process was implemented using an Excel spreadsheet developed in accordance with the Cochrane Consumers and Communication Review Group’s data extraction template. The spreadsheet enabled a systematic evaluation of the inclusion and exclusion requirements for all the selected studies. The extraction process was conducted independently by two authors (D.G-D and A.M-V.), with any disagreements being resolved through discussion until consensus was reached. A full record was kept of all articles that were not included, including the particular reasons for exclusion. The data were systematically recorded and stored in a spreadsheet.

### 2.6. Assessment of Study Methodology

The Physiotherapy Evidence Database (PEDro) scale was utilized to evaluate the methodological quality of pre-test and post-test studies with experimental (EXP) and control (CON) groups that were randomly selected. The methodological quality of each RCT was obtained from the PEDro database when available. For studies not listed in PEDro, two authors (D.G-D and C.D.G.-C.) independently assessed their quality, resolving any disagreements through consensus. The scale employs a range of 0 (low methodological quality) to 10 (high methodological quality) to score the internal study validity. The score that each section is awarded can range from 0 (“no”) to 1 (“yes”), depending on the quality obtained by each point. The quality of the studies were categorized according to the following cut-off points: excellent (9–10), good (6–8), fair (4–5), and poor (<3) [19]. The scale in question comprises ten items (see the Table 2).

## 3. Results

**Figure 1:**
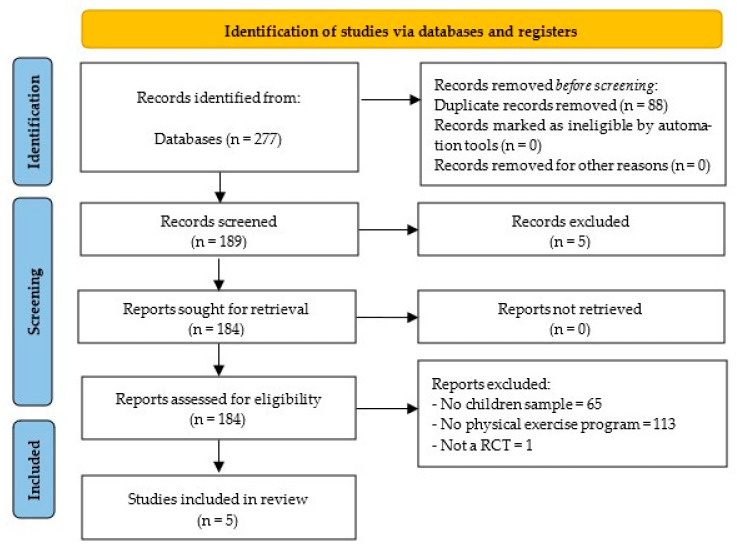
PRISMA flow diagram.

After analyzing all databases (FECYT: 5; PubMed: 10; SPORTDiscus: 2; ProQuest Central: 2; SCOPUS: 146; Cochrane Library: 112), the contents of 277 articles were checked, detecting, at the initial stage, 88 duplicate articles. Then, the authors analyzed whether each of the remaining 189 articles met all the inclusion criterion, resulting in the elimination of 183 articles by exclusion criterion number one (n = 65), exclusion criterion number two (n = 113), exclusion criterion number four (n = 1), and exclusion criterion number six (n = 5). The remaining five articles were included in the qualitative synthesis of the systematic review (Figure 1).

### 3.1. Methodological Quality

The quality assessment for this systematic review can be found in Table 2. Of the five studies included in this review, four were rated as fair [20,21,22,23], whereas the study by Cho et al. [24] was rated as good.

### 3.2. Study Characteristics

Five RCTs were included in this systematic review, analyzing the effect of physical exercise on BDNF levels in children up to 12 years of age [20,21,22,23,24]. The total sample comprised 385 participants, with individual study sizes ranging from 30 to 99 children. All interventions were conducted in clinical or educational settings and implemented structured physical activity programs lasting from 8 to 20 weeks, with training frequencies ranging from two to five sessions per week. Two studies enrolled healthy participants [23,24], two others recruited individuals with overweight or obesity [21,22], and one study involved participants with dysgraphia [20]. Only two of the five included studies [21,22] provided a detailed description of the control group activities, whereas the remaining three studies did not report this information.

Among the included studies, four assessed BDNF in serum [20,21,23,24], and one used plasma [22]. Most studies employed ELISA kits—particularly the Quantikine^®^ ELISA from R&D Systems—while Rodríguez-Ayllón et al. [22] used a Luminex-based multiplex immunoassay.

Exercise modalities included concurrent (aerobic and strength) training [21,22], Taekwondo [23,24], and motor skill-focused activities [20]. Interventions lasted 12–20 weeks and were delivered 3–5 days per week, and each session ran 45–90 min. Training intensity was monitored with heart rate targets in three studies [21,22,23] and by ratings of perceived exertion (RPE) in the trial by Cho et al. [24]; it was not objectively controlled in the remaining study.

### 3.3. Main Results

Two studies reported significant increases in BDNF following the intervention [20,24]. Ghafori et al. [20] observed a marked and statistically significant increase in serum BDNF levels after 12 weeks of combined fine and gross motor training in children with dysgraphia. Cho et al. [24] reported a significant rise in BDNF levels after 16 weeks of Taekwondo training in healthy children (*p* < 0.05). In both trials, the intervention groups showed significantly higher post-intervention BDNF levels than their respective control groups.

In contrast, the remaining three studies found no significant differences in BDNF levels post-intervention [21,22,23]. Kim et al. [23] found a trend toward increased BDNF after a 12-week Taekwondo-based exercise program (*p* = 0.066), although this result did not reach statistical significance. However, a significant increase in NGF was observed in that study (*p* < 0.001). On the other hand, Plaza-Florido et al. [21] and Rodríguez-Ayllón et al. [22]—both with longer, 20-week multicomponent interventions in children with overweight/obesity—reported no changes in BDNF levels after exercise (*p* > 0.05), nor any genotype × intervention interaction (in the case of Plaza-Florido et al. [21]).

From a quantitative perspective, two out of five studies (40%) reported a significant effect of exercise on BDNF levels [20,24]. These studies were characterized by sample sizes ≤ 40, training frequencies of ≥3 sessions per week, and durations of at least 12 weeks. By contrast, the two studies that enrolled children with overweight or obesity found no significant changes in BDNF levels [21,22]. The characteristics of the studies were extracted and clustered into Table 3.

## 4. Discussion

The present systematic review was conducted to address a significant gap in the pediatric exercise literature by focusing exclusively on RCTs that examined the effects of physical exercise interventions on BDNF levels in children aged 5 to 12 years. In contrast to previous reviews that combined data from both children and adolescents [15,16] or included studies with various designs, this is, to the best of our knowledge, the first review to concentrate solely on RCTs conducted in a pediatric population. These findings may be particularly valuable for coaches, physical education teachers, and other professionals responsible for designing and implementing training programs for young children. These insights may prove valuable for coaches, physical education teachers, and other professionals who design and deliver training programs for young children.

### 4.1. Exercise Modality and BDNF Response

The type of the exercise intervention has been identified as a significant factor influencing the BDNF response. The research utilizing neuromotor-oriented activities or martial arts programs, such as Taekwondo, has reliably shown beneficial effects on BDNF levels [20,23,24]. This observation is consistent with recent findings suggesting that physically engaging activities that also stimulate cognition may offer enhanced neuroplastic advantages relative to conventional aerobic exercise alone. The intricate motor patterns, balance challenges, and cognitive requirements of martial arts training may enlist multiple neural pathways, possibly provoking enhanced BDNF synthesis and release.

This pattern is consistent with recent meta-analytic evidence demonstrating that high-intensity exercise induces substantially greater BDNF responses than light-intensity protocols in adults [13], and network meta-analyses demonstrate that multifaceted training modes are most highly ranked for BDNF improvement [25]. The neural complexity and metabolic demands of activities like Taekwondo may potentially optimize BDNF responses through several mechanisms.

Ghafori et al. [20] provided evidence of a significant BDNF response following a 12-week combined fine and gross motor training program in dysgraphic children. The significant increase was also concomitant with significant improvements in executive functioning, implying a functional connection between the exercise-induced increase in BDNF and cognition improvement. This intervention’s particular focus on motor skill development may have optimized changes in neuroplasticity by directly stimulating the motor cortex and related brain networks.

Likewise, the research on Taekwondo [23,24] revealed beneficial BDNF responses. Cho et al. [24] observed impressive changes after 16 weeks of a training program. The multifaceted components of Taekwondo training that encompass cardiovascular endurance, strength building, flexibility, balance, and intricate motor skill learning could be offering a more composite stimulus to neurotrophin production compared to the conventional types of exercise. In this regard, Roh et al. [26] showed a significant increase in BDNF following a 16-week Taekwondo program in overweight and obese adolescents.

### 4.2. Population-Specific Responses

Interestingly, studies on healthy children have consistently reported positive BDNF responses, while those focusing on children with obesity or overweight have shown no significant changes [21,22]. This divergent response pattern suggests that metabolic status may influence the control of exercise-induced BDNF. Moreover, obesity in children is associated with chronic low-grade inflammation, insulin resistance, and impaired neurotrophic factor signaling, which may attenuate the normal BDNF response usually induced by exercise.

This finding is supported by research demonstrating that obesity creates a state of chronic inflammation with elevated cytokines, including IL-6, TNF-α, and C-reactive protein [27], which may interfere with BDNF signaling pathways and diminish exercise-induced neuroplastic responses. Meta-analytic evidence shows that exercise training can significantly reduce these inflammatory markers in children and adolescents [28], suggesting that anti-inflammatory effects may be a prerequisite for optimal BDNF responses in obese populations.

Plaza-Florido et al. [21] and Rodríguez-Ayllón et al. [22] conducted long-term 20-week multicomponent interventions in overweight and obese pediatric populations; however, both studies did not find any statistically significant changes in BDNF levels. The authors suggested that increased exercise intensities or longer intervention durations may be needed to reverse the metabolic dysfunctions associated with excessive adiposity. Modern research supports this postulate, showing that while acute physical activity increases circulating BDNF levels in obese adults, long-term exercise training programs often cannot promote sustained elevations because of the existence of inflammatory states and metabolic dysregulation [29]. Similarly, Roh et al. [26] reported a significant increase in BDNF following a 16-week Taekwondo program in overweight and obese adolescents. However, no long-term follow-up was conducted to assess the sustainability of this effect.

### 4.3. Frequency and Duration of Intervention

The analysis showed that effective interventions generally lasted 12–20 weeks, with training frequencies of three–five sessions per week and session durations of 45–90 min. This is relevant because previous studies have suggested that session length can influence acute BDNF responses [30]. Our finding aligns with adult research indicating that chronic exercise adaptations—including neuroplastic changes—require sustained training stimuli over several months. Moreover, recent meta-analytic evidence confirms that both acute and long-term physical exercise significantly raise circulating BDNF levels, with optimal responses achieved at training frequencies of two–three sessions per week [31].

The standard frequency threshold of at least three sessions per week aligns with children’s recommended levels of physical activity [12] and signals that frequent and regular exercise is paramount with a view to maintaining heightened levels of BDNF. Such frequency can perhaps be indispensable with a view to maintaining the cellular signaling pathways (PI3K/Akt, MAPK/ERK, and PLC-γ) responsible for BDNF synthesis and its secretion [1].

Recent meta-analyses show that circulating BDNF increases acutely after a single session of structured exercise, whereas extended training programs do not consistently increase circulating BDNF concentrations [29,32,33]. However, studies with larger sample sizes and follow-up periods of at least six months are needed to confirm this issue in the present sample.

### 4.4. Biological Matrix and Measurement Considerations

Most of the studies (four out of five) measured BDNF in serum using ELISA methods, while one study used plasma through a Luminex-based multiplex immunoassay [22]. The choice of biological matrix and analytical method could influence the results since serum BDNF levels are typically higher than in plasma due to degranulation of platelets during the coagulation procedure. Platelets serve as the major peripheral reservoir for BDNF, and activation during blood collection can significantly influence the concentrations measured [34].

Extending the blood coagulation time to 30–60 min can substantially elevate BDNF levels [33]. Given that circulating BDNF levels typically return to baseline within a few hours post-exercise [35,36], the variability in sample collection timing across studies likely contributed to the heterogeneity of the results. Additional discrepancies may stem from differences in analytical methods, assay kits, sample processing protocols, and participant characteristics. This methodological heterogeneity underscores the urgent need for standardized protocols in future research to improve the comparability and interpretability of findings across studies.

### 4.5. Mechanism Considerations

The different responses observed across exercise modalities and populations provide insights into potential mechanisms underlying exercise-induced BDNF regulation. Neuromotor activities and martial arts training engage multiple brain regions simultaneously, including motor cortex, cerebellum, prefrontal cortex, and limbic structures. This widespread neural activation may provide a more potent stimulus for BDNF synthesis compared to repetitive aerobic activities.

Recent mechanistic insights show that exercise-induced upregulation of BDNF is mediated by β-hydroxybutyrate, a ketone body produced during sustained physical activity, which acts as an endogenous inhibitor of histone deacetylases to increase activity-dependent BDNF gene transcription [34]. This mechanism could potentially explain the requirement of long exercise intervention periods (≥12 weeks) for appreciable BDNF adaptations in children.

The interaction between exercise intensity and BDNF responses supports that metabolic stress may provide a critical stimulus for neurotrophin upregulation, with exercise intensities leading to accumulation of blood lactates as especially important with respect to stimulating BDNF responses [37]. This can help explain why complex high-intensity practices like martial arts produce larger responses compared with multicomponent, moderate-intensity training programs.

### 4.6. Practical Applications and Clinical Implications

Structured physical activity programs that incorporate complex motor skills, martial arts training, or neuromotor activities may be effective in elevating BDNF levels. To elicit meaningful neurobiological adaptations, such interventions should be implemented at a minimum frequency of three sessions per week and sustained for at least 12 weeks.

In an educational setting, the integration of physically challenging activities that challenge cognitive processes could bring about twofold benefits in the areas of physical fitness and cognitive function. For example, the correlation established by Ghafori et al. [20] between increased BDNF levels and improvements in executive function suggests that specific motor-based interventions may serve as effective adjunct therapies for children experiencing learning difficulties.

Conventional multicomponent exercise protocols may not be enough to evoke BDNF responses in overweight/obese children. However, this issue has only been addressed in two RCTs to date. The different responses between healthy and obese children would suggest that population-specific exercise prescriptions would be necessary to optimize neuroplastic effects.

## 5. Limitations and Future Research Directions

Despite the novelty of the present review, it is important to acknowledge several limitations in the interpretation of its findings. First, the review includes only five studies with a total of 385 participants. This small number of studies and limited sample size substantially restrict the generalizability of the findings and preclude the possibility of conducting a meta-analysis. Moreover, considerable heterogeneity exists among the included studies in terms of participant characteristics (e.g., country, sex, or health status), intervention protocols, outcome measures, and evaluation tools. This diversity complicates direct comparisons and likely contributes to the mixed findings reported.

A major methodological limitation is the lack of standardized protocols for BDNF measurement. Variability in biological matrices (serum vs. plasma), analytical methods (e.g., different ELISA kits vs. multiplex assays), and sample handling procedures likely accounts for the wide range of baseline BDNF levels observed across studies. Future research should prioritize the development and adoption of standardized measurement protocols to enable meaningful comparisons and support meta-analytic approaches.

The different effects observed between healthy children and their overweight/obese peers imply significant group-specific considerations that merit further investigation. Future studies should assess dose–response relationships across a range of pediatric populations, determining whether greater exercise intensity, longer intervention durations, or multifaceted lifestyle interventions are necessary to elicit BDNF responses in children with metabolic syndrome.

Long-term follow-up investigations are necessary to ascertain the duration of exercise-induced changes in BDNF and how they relate to cognition and academic achievement. Additionally, mechanistic investigations examining the neurobiological processes underlying exercise-induced BDNF regulation in children would yield essential data for tailoring intervention protocols and clarifying variability in response profiles. Given the limited number of available studies, another promising line of research would be to investigate the effects of various types of exercise programs, such as team sports, yoga for children, aquatic exercise, or game-based interventions, on BDNF levels and related cognitive benefits.

## 6. Conclusions

This systematic review provides preliminary evidence that structured exercise interventions may enhance BDNF levels in children aged 5 to 12 years. However, the results of the analyzed studies were mixed. Only two out of the five included studies reported an acute increase in BDNF levels compared to baseline or control groups.

The findings suggest that exercise modality, population characteristics, and intervention parameters are key determinants of BDNF responsiveness. For instance, martial arts programs delivered at a frequency of three or more sessions per week over a period of at least 12 weeks may be effective in eliciting acute increases in BDNF among children. Conversely, children with overweight or obesity may require longer or more intensive interventions to achieve similar neurobiological benefits.

Although the current evidence base remains limited, these results are consistent with broader efforts to integrate cognitively engaging physical activity into educational and clinical settings, aiming to support brain health and cognitive development during a critical period of maturation. Future research should prioritize the use of standardized BDNF measurement protocols, larger and more representative pediatric samples, and intervention designs tailored to specific subgroups within the child population.

## Figures and Tables

**Table 1:** Inclusion and exclusion criteria.

Item	Inclusion	Exclusion	Search Coherence
Population	School-aged children	Children out of school age Children under medical treatment	preschool* OR kindergarten OR child* OR young OR childhood OR school
Intervention or Exposure	Children doing exercise or physical activity	Population from other age range. Children not doing exercise or physical activity. Interventions where other factor is implemented (e.g., supplementation, transcranial stimulation) Study protocols Adolescents receiving pharmacological treatments	exercise OR movement OR activity OR sport OR fitness OR aerobic OR training OR performance
Comparation	-	-	
Outcome[s]	Outcomes related to brain-derived neurotrophic factor	Outcomes not related to brain-derived neurotrophic factor	BDNF OR “brain-derived neurotrophic factor”
Design	Randomized controlled trial	Non-randomized controlled trials	“randomized controlled trial”
Other criteria	Peer-reviewed full-text studies published in original journal articles	Non-peer reviewed journal articles. Non-original full-text studies (conference papers…).	

**Table 2:** Methodological assessment of the included studies.

	Ghafori et al. [20]	Plaza-Florido et al. [21]	Rodriguez-Ayllon et al. [22]	Kim et al. [23]	Cho et al. [24]
Subjects were randomly allocated to groups.	1	1	1	1	1
Allocation was concealed.	0	0	1	0	0
The groups were similar at baseline regarding the most important prognostic indicators.	1	1	1	1	1
There was blinding of all subjects.	0	0	0	0	0
There was blinding of all therapists who administered the therapy.	0	0	0	0	0
There was blinding of all assessors who measured at least one key outcome.	0	0	0	0	0
Measures of at least one key outcome were obtained from more than 85% of the subjects initially allocated to groups.	0	0	0	1	1
All subjects for whom outcome measures were available received the treatment or control condition as allocated or, where this was not the case, data for at least one key outcome was analyzed by “intention to treat”.	0	0	0	1	1
The results of between-group statistical comparisons are reported for at least one key outcome.	1	1	1	1	1
The study provides both point measures and measures of variability for at least one key outcome.	1	1	1	1	1
SCORE	4	4	5	5	6
	Fair	Fair	Fair	Fair	Good

**Table 3:** Main characteristics and findings about the effects of exercise on adolescents’ brain-derived neurotrophic factor.

Ref.	Participants	BDNF Registration	Other Criteria to Consider	Exercise Information	Results	Conclusions
Ghafori et al. [20]	N = 40 males (10.4 ± 3.5 years) Country: Iran With diagnosed dysgraphia Mean intelligence quotient: 78.81 ± 3.78	Biological matrix: Serum ELISA method (Quantikine^®^ R&D Systems, kit #DBD00). Fasting sample collection, 48 h before and after the procedure. Venous extraction (5 cc), with processing by centrifugation at 3000 rpm for 10 min at 4 °C.	Pre-screening by interview, child symptom inventory (parent/teacher version), RCPM and apraxia index by block test. Exclusion of other relevant psychological or motor disorders.	IG: 12 weeks (3 sessions/week of 45 min each session) Three types of exercise (each for 10–15 min). Exercises comprising fine (e.g., cutting and sticking colored paper, Frostig exercises, targeted exercises) and gross motor exercises (e.g., throwing various types of balls; catching; dribbling; passing; walking on a spiral path while bouncing the ball off the ground) Intensity: Exercises were arranged from easy to difficult CG: NR	IG showed a significant increase in BDNF *** (from 542.47 ± 5.08 to 642.80 ± 21.41 pg/mL). Significant increase in BDNF serum levels in IG compared with CG after intervention. Significant reduction in perseverative errors (18.81 → 15.50) and total errors (35.56 → 27.13) in IG. Negative correlation between BDNF and errors (r = −0.445 for perseverative errors; r = −0.461 for total errors). Fifty-two percent of the variance in perseverative errors and 39% in total errors were explained by BDNF level.	Twelve weeks of motor exercise significantly improve both serum BDNF level and executive function in children with dysgraphia. The increase in BDNF could be a neurophysiological mechanism associated with cognitive improvements. The usefulness of structured motor interventions in clinical and school contexts for this population with specific educational needs is highlighted.
Plaza-Florido et al. [21]	N = 99 (57 males and 42 females; 10.03 ± 1.51 years) Country: Spain ActiveBrains clinical trial participants Overweight or obese	Biological matrix: Serum ELISA method (kit Human BDNF Quantikine^®^, R&D Systems, Cat# DBD00).	The BDNF Val66Met (rs6265) polymorphism was analyzed using DNA extracted from peripheral blood (Puregene Kit, QIAGEN). Genotype × intervention interactions on BDNF levels were analyzed.	IG: 20 weeks (3–5 sessions/week of 90 min each session) Concurrent exercise: - Aerobic: 60 min - Strength: 30 min Average intensity of 38 min per session at >80% HRmax. CG: Continued their usual routines	No significant differences in serum BDNF levels in the IG (1552.26 ± 387.87 → 1552.31 ± 472.26 pg/mL). No significant interactions between Val66Met genotype and exercise response were observed in BDNF (*p* = 0.281) No effects of exercise on BDNF were reported when subgroups were analyzed by sex, age, or pubertal maturation.	The 20-week multicomponent exercise program did not produce changes in serum BDNF levels in overweight/obese children. These results suggest that, in this pediatric population, BDNF may not be sensitive to interventions of this type or that higher doses or longer durations are required to generate measurable effects.
Rodriguez-Ayllon et al. [22]	N = 81 (48 males and 33 females; 10.12 ± 1.11 years) Country: Spain ActiveBrains clinical trial participants Overweight or obese	Biological matrix: Plasma. XMap method (Luminex) with EMD Milliplex Map Kit panel (Millipore).	Other candidate biomarkers: β-hydroxybutyrate (BHB) by colorimetry; Cathepsin B (CTSB), FGF21, and Kynurenine by ELISA; sVCAM-1 by XMap. Exploratory biomarkers: 92 neurological proteins by proximity extension assay (Olink Bioscience).	IG: 20 weeks (3–5 sessions/week of 90 min each session) Concurrent exercise: - Aerobic: 60 min - Strength: 30 min Average intensity of 38 min per session at >80% HRmax. CG: Continued their usual routines	There was no significant effect (*p* > 0.05) on BDNF or BHB, CTSB, kynurenine, FGF21, or sVCAM-1. Significant reduction in 6 neurological proteins (CPA2, KYNU, LAIR2, MSR1, PLXNB3, SCARB2), although only MSR1 maintained significance after correction for FDR ***. There was no protein mediator between exercise and brain health outcomes.	No chronic effects of exercise on candidate biomarkers related to brain health were found. However, a consistent reduction in MSR1 was observed, potentially relevant as a new biomarker in future research. Further investigation of the effects of chronic exercise on MSR1 and other markers, as well as its relationship to brain health in overweight or obese pediatric populations is recommended.
Cho et al. [24]	N = 30 (18 males and 12 females; 11.20 ± 0.77 years) Country: South Korea Without pathologies	Biological matrix: Serum ELISA method (BDNF Quantikine Kit, Cat# DBD00, R&D Systems). Other biochemical variables: VEGF and IGF-1 also analyzed by ELISA.	Cognitive function parameters (Stroop test) and cerebral blood flow velocity (CBF) were evaluated by transcranial Doppler. VO_2_max was measured with modified Balke protocol.	IG: 16 weeks (5 sessions/week of 60 min each session) Content of the sessions: - General physical training (shuttle run, Burpee, vertical jump, etc.). - Basic Taekwondo movements - Poomsae (Taegeuk forms 1–8) - Kicking and displacement techniques - Taekwondo-based gymnastics Average intensity of 11–15 RPE CG: NR	Significant increase * in BDNF values only in the IG (Pre: 24.03 ± 6.16 ng/mL → Post: 27.62 ± 7.58 ng/mL). Significant increase in BDNF serum levels in IG compared with CG after intervention. VEGF and IGF-1 values were also significantly increased in IG alone. Significant increase in VEGF and IGF-1 in IG compared with CG after intervention. No significant changes were observed in MCAs, MCAd, MCAm, or PI (*p* > 0.05). Cognitive function: Significant improvement *** in cognitive function, specifically in color–word Stroop subtest score in the IG compared to pre-intervention and CG.	Taekwondo training for 16 weeks caused a significant increase in serum levels of BDNF, VEGF, and IGF-1 in healthy children, suggesting an activation of neuroplastic mechanisms similar to those of aerobic exercise. Although no changes in cerebral blood flow were observed, exercise could indirectly modulate brain health through increased growth factors.
Kim et al. [23]	N = 30 males (10.93 ± 0.26 years) Country: South Korea Without pathologies	Biological matrix: Serum ELISA method (Human BDNF Quantikine^®^, R&D Systems, Cat# DBD00). Additional comparison: NGF also analyzed with ELISA (Abnova, Cat# KA0399).	Working memory was measured using the K-WISC-III test (digits forward and digits backward subtests). Complete physical fitness assessment: cardiovascular endurance, strength, flexibility, speed and agility.	IG: 12 weeks (5 sessions/week of 60 min each session) Content of the sessions: Progressive exercises per week, including jumping, hexagonal ladder, pull-ups, sit-ups, kickboxing, balance board exercises, plank, Taekwondo-type musical circuits. Average intensity of 50–80% HRmax CG: NR	Increase in BDNF values in IG post-intervention (31.74 ± 5.46 to 34.32 ± 3.21 ng/mg; *p* > 0.005). Significant *** increase in NGF values in IG (33.12 ± 3.32 → 39.15 ± 2.70 pg/mL). Significant increase in NGF in IG compared with CG after intervention. No significant changes (*p* > 0.05) were observed in parameters related to working memory. Significant improvements * in cardiovascular endurance, strength and agility in the exercise group.	Although the 12-week Taekwondo-based intervention significantly improved NGF and several physical abilities in male schoolchildren, no statistically significant changes in BDNF or working memory were observed. Programs that integrate cognitive tasks or learning during the session could be more effective in inducing brain functional improvements.

Note: BDNF: brain-derived neurotrophic factor; BMI: body mass index; CG: control group; HRmáx: heart rate maximum; IG: intervention group; IGF-1: insulin growth factor-1; IGFBP-3; NGF: nerve growth factor; ng/mL: nanograms per milliliter; pg/mL: picograms per milliliter; NR: not reported; RPE: rate of perceived exertion; VO_2_max: maximum oxygen volume. Statistical significance: * *p* < 0.05, *** *p* < 0.001.

## Data Availability

Data are contained within the article.
